# Large scale analysis of positional effects of single-base mismatches on microarray gene expression data

**DOI:** 10.1186/1756-0381-3-2

**Published:** 2010-04-29

**Authors:** Fenghai Duan, Mark A Pauley, Eliot R Spindel, Li Zhang, Robert B Norgren

**Affiliations:** 1 Center for Statistical Sciences, Brown University, Providence, RI, USA; 2 College of Information Science & Technology, University of Nebraska at Omaha, Omaha, NE, USA; 3 Division of Neuroscience, Oregon National Primate Research Center, Oregon Health & Science University, Beaverton, OR, USA; 4 Department of Bioinformatics and Computational Biology, University of Texas MD Anderson Cancer Center, Houston, TX, USA; 5 Department of Genetics, Cell Biology and Anatomy, University of Nebraska Medical Center, Omaha, NE, USA

## Abstract

**Background:**

Affymetrix GeneChips utilize 25-mer oligonucleotides probes linked to a silica surface to detect targets in solution. Mismatches due to single nucleotide polymorphisms (SNPs) can affect the hybridization between probes and targets. Previous research has indicated that binding between probes and targets strongly depends on the positions of these mismatches. However, there has been substantial variability in the effect of mismatch type across studies.

**Methods:**

By taking advantage of naturally occurring mismatches between rhesus macaque transcripts and human probes from the Affymetrix U133 Plus 2 GeneChip, we collected the largest 25-mer probes dataset with single-base mismatches at each of the 25 positions on the probe ever used in this type of analysis.

**Results:**

A mismatch at the center of a probe led to a greater loss in signal intensity than a mismatch at the ends of the probe, regardless of the mismatch type. There was a slight asymmetry between the ends of a probe: effects of mismatches at the 3' end of a probe were greater than those at the 5' end. A cross study comparison of the effect of mismatch types revealed that results were not in good agreement among different reports. However, if the mismatch types were consolidated to purine or pyrimidine mismatches, cross study conclusions could be generated.

**Conclusion:**

The comprehensive assessment of the effects of single-base mismatches on microarrays provided in this report can be useful for improving future versions of microarray platform design and the corresponding data analysis algorithms.

## Introduction

High-density microarrays have revolutionized biomedical research by providing comprehensive profiling of DNA and RNA molecules extracted from normal and diseased cells and tissues [1]. The Affymetrix GeneChip is one of the most frequently used microarray platforms for gene expression and genotyping assays [1,2]. For gene expression assays, each transcript is assessed by a set of 25-mer oligonucleotides probe pairs, a Perfect Match (PM) probe and a Mismatch (MM) probe; the difference between the PM probe signals and MM probe signals are used to estimate gene expression levels. In genotyping assays, PM/MM probe pairs are used to determine SNP calls. The MM probe is identical to the PM with the exception that there is a mismatch nucleotide at position 13, i.e., the center position of the probe http://www.affymetrix.com. Upon binding to the targeted molecule, the mismatch is expected to cause a disruption around position 13 and destabilize the binding. Consequently, a MM probe should yield less signals than the corresponding PM probe. MM probes were designed to measure nonspecific binding and background noise. However, the observed MM probe signals are sometimes substantially higher than the corresponding PM probe signals, which is unexpected from microarray design [3,4]. Clearly, it is important to assess the effects of mismatches on probe signal intensity and the subsequent consequences in the interpretation of the microarray data. There have been several previous studies focused on the issue [5-11], which revealed that the hybridizations on microarrays are much more complicated than hybridization in solution. It was found that the mismatch effects may depend on the mismatch types, the positions on the probe, florescent labels and off-target hybridizations. The exact mechanisms remain unclear. Clearly, more data is necessary to understand the effects of mismatches on miocrarrays.

In this study, we hybridized rhesus macaque mRNA samples with arrays designed for the human genome (Human Genome U133 Plus 2 [Affymetrix, Inc., California]) and with arrays designed for the rhesus genome (Rhesus GeneChip [Affymetrix]). By comparing species differences in target sequences, we were able to examine the effects of a large number of mismatches (15,800) between target and probe on signal intensity. Moreover, the mismatch positions in our dataset are not limited to the center of the probes, which allowed us to perform the most in-depth analysis done to date of the effect of mismatches at each of the 25 probe positions.

## Materials and methods

### Microarray Data

Rhesus macaque RNA samples from five sources (immortalized fibroblast, cerebral cortex, pancreas, testes and thymus) were equally divided into two sets of aliquots. Samples from each of the five sources were labeled and hybridized with either two Rhesus Macaque Genome microarrays (Affymetrix) or two Affymetrix human genome microarrays (HGU133plus2.0) according to the manufacturer's instructions. Thus, a total of 10 rhesus and 10 human microarrays were processed. See Duan et al. 2007 for further details [12].

### Affymetrix GeneChip Preprocessing

**Figure 1:**
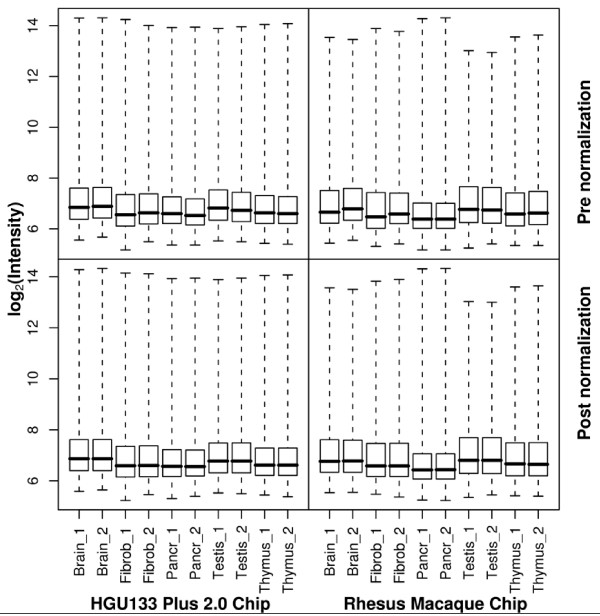
**Probe-level log intensities for 10 HGU133 plus 2.0 Microarrays and 10 rhesus macaque Microarrays**. Each tissue type was hybridized with two human and two rhesus GeneChips. The two upper plots were generated from raw data (before normalization) and the two lower plots were generated from data after quantile normalization was performed.

The .CEL files were exported and raw PM intensities were extracted for the 20 arrays. Quantile normalization was applied within two array replicates to control for variation in hybridizations (Figure 1). The raw data (20 .CEL files) have been uploaded to the GEO repository [13] (GEO accession no. GSE9531).

### Identifying Single Base-Pair Mismatches between Human and Rhesus Probes

A five-step procedure was used to identify single base-pair mismatches between human and rhesus probes contained on their respective microarrays. Step 1: Rhesus macaque consensus sequences that corresponded to probesets from the HGU133plus2.0 GeneChip were obtained from Affymetrix. Step 2: All of the HGU133Plus2 probes in a given probeset were aligned with the corresponding rhesus consensus sequence using the *GAP *package in Accelrys GCG http://www.accelrys.com. A GAPweight of 50 and a LENgthweight of 0.5 were used for the alignment. Scripts written in the Python programming language were used to submit alignments to GAP and parse the resulting output. Results from this step were stored in a relational database. Step 3: Probesets for which the probes did not align sequentially with the consensus sequence and those for which the alignment was incomplete were discarded. Step 4: 15,800 HGU133plus2.0 probes (from approximately 12,500 probesets) for which there was a single mismatch between the HGU133Plus2 probe and the rhesus consensus sequence in each of positions 1 to 25 were identified via database queries. These probes were used as MM probes in the MM to PM comparison that followed. Step 5: The *matchprobes *package in Bioconductor was used to search for the rhesus probe sequences that corresponded to the human probes identified in Step 4 [14]. The probes identified in this way were used as PM probes in the MM to PM comparison. The *matchprobes *package was also used to identify a set of 36,578 probes (from approximately 22,000 probesets) that were identical on the human and rhesus microarrays for use as controls.

### Statistical Analysis

The log-scaled ratios of PM and MM_*i *_signals, i.e., log_2_(PM/MM_i_) where *i *is the position of the single-base-pair mismatch, were used to represent the differences of the signal intensities between PM and MM_*i *_probes. In other words, log_2_(PM/MM_i_) represents the discrimination of PM and MM at the *i*th position [11]. For each tissue (or cell line), log_2_(PM/MM_i_) was calculated as the mean value from the two replicates. The average of log_2_(PM/MM_i_) over each tissue (or cell line) was obtained by applying a one-way ANOVA to each probe with the variables of *Tissue *(five levels, representing the five sources of RNA described in the "Microarray Data" section) and *Chip *(two levels, representing PM and MM_*i*_).

To examine the effect of different types of mismatches at different mismatch positions, the estimates of log_2_(PM/MM_i_) from the last step were categorized according to mismatch type and mismatch position. Mismatch type was named so that the first letter denotes the base in PM and the second letter denotes the base in MM. The 25 mismatch positions were binned into 3 groups: the left group had mismatches in positions 1 to 8, denoted as the 5' end of a probe; the center group had mismatches in positions 9 to 17 and the right group had mismatches in positions 18 to 25, denoted as the 3' end of a probe. The statistical significance was assessed with Student's t-test.

## Results

We searched for pairs of probes that differed by a single nucleotide on the human U133Plus2 array and the rhesus macaque GeneChip. 15,800 such probe pairs were found. Table 1 shows the frequency of probes with mismatches at different positions. At least 500 probe pairs were found for each of the 25 positions. In general, there were fewer mismatch probes in the center positions than in the 5' and 3' ends. Position 13 had the fewest number of mismatch probes.

**Table 1:** Frequency of probe pairs for each mismatch position.

Single-base-pair mismatch position	Number of PM/MMi
1	689
2	614
3	678
4	644
5	590
6	672
7	667
8	639
9	682
10	587
11	596
12	533
13	521
14	540
15	547
16	559
17	642
18	633
19	630
20	622
21	686
22	667
23	652
24	763
25	747

**Figure 2:**
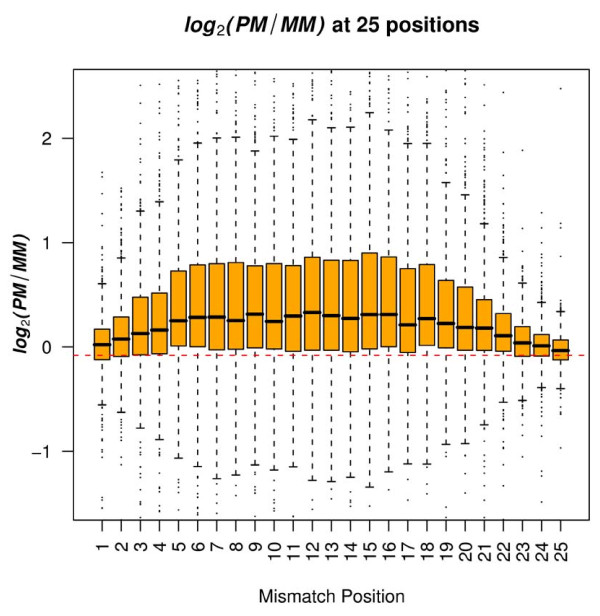
**Distribution of log_2_(PM/MM_i_) across 25 single-base-pair mismatch positions**. The red dashed line represents the mean value of log_2_(PM/MM_i_) when there was no mismatch, i.e., the common PM probes from the HGU133plus2.0 GeneChip and the Rhesus macaque GeneChip.

**Figure 3:**
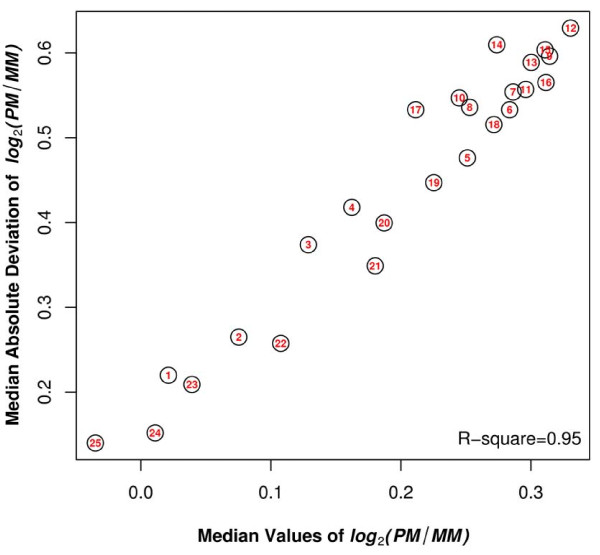
**Average of log_2_(PM/MM_i_) versus the variability of log_2_(PM/MM_i_) across 25 mismatch positions**. The values of the median and the median absolute deviation (MAD) of log_2_(PM/MM_i_), representing the robust versions of the average and variability, were calculated for each mismatch position. The number inside the circle indicates the mismatch position. R- square measures the strength of the linear relationship between two variables.

The average difference between PM and MM depended on the position of the mismatch on the probe. Both the average and variability (represented by the interquantile range) of log_2_(PM/MM_i_) increased from both ends of the probes (positions 1 and 25) towards the center, and became relatively steady between positions 7 - 16 (Figure 2). The peak value of the average of log_2_(PM/MM_i_) was reached when *i *was equal to 12. For example, there was a 24% decrease in the value of PM/MM when the position changed from 12 to 1, and a 29% decrease in the value of PM/MM when the position changed from 12 to 25. In addition, we observed a strong linear association between the average and the variability of log_2_(PM/MM_i_)*(*R^2 ^= 0.95) (Figure 3).

Because the PM signals and MM signals were collected from different arrays, it is possible that the difference between the signals resulted in part from different hybridization conditions on the two types of array. To test this, we compared signals from probes that matched perfectly between the human array and the macaque array. As expected, the average differences were very small as compared to values obtained from log_2_(PM/MM_i_) across 25 probe positions (Figure 2). The p values from Student's t-tests comparing log_2_(PM/MM_i_) with the signal differences of identical probe pairs between human and monkey microarrays were extremely small (<10^-6^).

**Figure 4:**
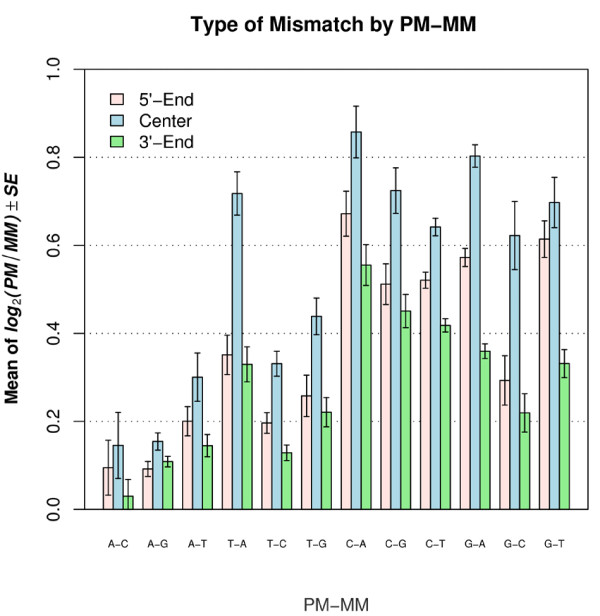
**Mean level of log_2_(PM/MM_i_) for 3 groups of mismatch positions for each mismatch type**. The x-axis denotes the 12 mismatch types. The first nucleotide represents the rhesus probe (PM) and the second nucletotide represents the human probe (MM).

We found that mismatch type also had a strong effect on signal intensity. The greatest effects were observed with C → A (i.e., C on PM and A on MM; correspondingly G on target), G → A and C → G. The least effects were observed with A → C, A → G and A → T (Figure 4). In general, mismatching in a C-G base pair resulted in greater loss of intensity than when mismatching occurred in an A-T base pair.

To determine whether different types of nucleotide mismatches differ significantly across mismatch positions, the mean level of log_2_(PM/MM) was calculated and graphed for different categories according to mismatch type and mismatch position classification (Figure 4). Note that the subscript from MM_*i *_was removed because the mismatch positions were binned into groups. The signal intensity difference between PM and MM was represented by the average of log_2_(PM/MM). This difference was always greater for the center group (positions 9 - 17) than either the 5' end (positions 1 - 8) or the 3' end (positions 18 - 25). On average, the discrimination between PM and MM for the center group was 13% higher than for the 5' end group and 17% higher than for the 3' end group. In addition, the value of log_2_(PM/MM) in the 5' end was greater, with the exception of C-A, than the 3' end. The differences between the center group and the other two groups were statistically significant in most cases (Table 2). Only 3 out of 24 comparisons had p values larger than 0.05 (Table 3). However, there were 8 of 12 comparisons with p value larger than 0.05 when the 5' end was compared to the 3' end (Table 2).

**Table 2:** Student's t-tests between 3 groups of mismatch positions for each mismatch type.

Mismatch type	Center vs. 5'-end	Center vs. 3'-end	5'-end vs. 3'-end	Frequency
A-C	0.00187	0.000128	0.525*	673
A-G	6.29E-05	6.78E-10	0.0423	2567
A-T	2.78E-08	8.31E-10	0.747*	553
T-A	0.0821*	0.00499	0.328*	506
T-C	0.00733	0.047	0.461*	2920
T-G	0.553*	0.185*	0.422*	435
C-A	0.187*	2.54E-09	1.89E-06	778
C-G	0.000115	3.10E-06	0.377*	627
C-T	1.47E-14	2.13E-46	1.66E-12	2874
G-A	2.17E-06	8.23E-19	6.08E-05	2624
G-C	0.00103	2.76E-05	0.339*	687
G-T	0.0126	5.62E-05	0.103*	556

*: p value ≥ 0.05

**Table 3:** Similarities across different studies in terms of the effect of mismatch type.

3a:					
	
	Spearman Correlation Coefficients, N = 12 (mismatch type) (Prob > |r| under H0: Rho = 0)				
	
	Pozhitkov2006	Schwarz2006	Wick2006	Naiser2008	Current Study
Pozhitkov2006	1.00000	0.60596 (0.0368)	-0.07005 (0.8287)	0.06655 (0.8372)	0.45884 (0.1335)
Schwarz2006		1.00000	-0.05594 (0.8629)	0.05594 (0.8629)	0.60839 (0.0358)
Wick2006			1.00000	0.62937 (0.0283)	0.33566 (0.2861)
Naiser2008				1.00000	0.56643 (0.0548)
Current study					1.00000
**3b:**					
	
	**Pearson Correlation Coefficients, N = 12 (mismatch type) (Prob > |r| under H0: Rho = 0)**				
	
	**Pozhitkov2006**	**Schwarz2006**	**Wick2006**	**Naiser2008**	**Current study**
**Pozhitkov2006**	1.00000	0.46958 (0.1235)	-0.11957 (0.7113)	-0.07138 (0.8255)	0.19784 (0.5377)
**Schwarz2006**		1.00000	-0.14616 (0.6503)	-0.01975 (0.9514)	**0.59298** (0.0421)
**Wick2006**			1.00000	**0.71084** (0.0096)	0.47112 (0.1221)
**Naiser2008**				1.00000	**0.57443** (0.0508)
**Current study**					1.00000

3a: using Spearman's correlation coefficient; 3b: using Pearson's correlation coefficient. The statistics in bold indicate the correlation coefficient significantly different from the chance, i.e., p value ≤ 0.05; or marginally significantly different, i.e., 0.05 < p value < 0.07.

## Discussion

We took advantage of the differences between the human and the rhesus macaque genomes to evaluate the effects of mismatches between targets and probes on signal intensity in expression arrays using the largest dataset studied thus far. In a previous study, the positional effect of single base mismatches collected data from 935 pairs of perfect match and mismatch probes were examined [11]. Our study utilized data from 15,800 pairs of perfect match and mismatch probes.

### Nonrandom Number of Mismatch Probes by Position

We expected to find approximately the same number of mismatches between human probes and rhesus sequences at all 25 mismatch positions. However, there was a general tendency for fewer mismatch probes at central positions than at the 5' and 3' ends. Interestingly, we found the fewest mismatches between human probes and monkey sequence at position 13. The explanation for this result is not obvious. One possibility is that the human probes were selected by Affymetrix to have fewer mismatches in central regions due to human SNPs. This preference for relatively conserved regions may have resulted in fewer probes with mismatches to rhesus sequence in this central region.

### Mismatch Positional Effects

We found a mismatch at the center of a probe resulted in a greater loss in signal intensity than a mismatch at the ends of the probe. Various studies reported similar results [5-7,11]. This may be due to the zippering effect, as discussed by Binder [15] and Deutsch et al [16]. The probability of "open" base-pairings may be minimal in the center of an oligonucleotide, on average, and thus the discriminating effect of the central bases may be maximal.

We also observed a slight asymmetry between the ends of a probe: the effects of mismatches at the 5' end of a probe were greater than those at the 3' end. This may be due to the fact that the probes on the Affymetrix microarrays are anchored to the silica surface at the 3' end of the probe. Thus, our result is consistent with Wick et al.'s finding that mismatches at the loose end are more discriminating than mismatches at the attached end.

Our results also suggested that the stronger the mismatch effect, the more variable the effect. In Figure 3, we showed that the value of log_2_(PM/MM_i_) is closely correlated with the median absolute deviation value of log_2_(PM/MM_i_). This may be because when the mismatch is strongly disruptive of the binding interaction on the probe, the adjacent nucleotides will have to accommodate the disruption. Thus, certain combinations of nucleotides may be more accommodating than others, leading to more variation in the free energy of binding for the more disruptive mismatch types.

### Mismatch Type Effects

**Figure 5:**
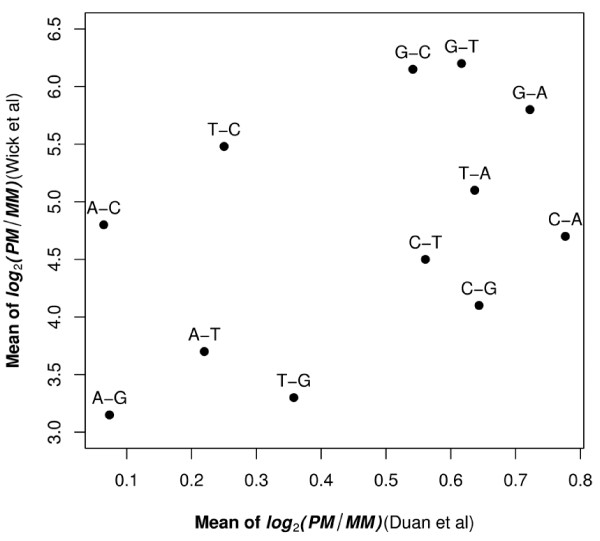
**Comparison of log_2_(PM/MM_i_) for each mismatch type between the current study and Wick et al**. For each mismatch type, the first nucleotide represents the PM and the second nucleotide represents the MM.

The role of the probe mismatch type on signal intensity has been previously investigated [6,7,9-11]. Among these studies, Wick et al's report (2006) and our analysis used the same statistic - log_2_(PM/MM) - to measure the discriminating ability of PM and MM. The biggest difference between our results and Wick et al's was the magnitude of PM/MM (Figure 5). On average, Wick et al's reported a 20-fold greater effect than was observed in the current study. This likely reflects the fact that the length of the probe was 18 oligonucleotides in Wick et al's report and 25 oligonucleotides in our study because different arrays were used in the two studies.

**Figure 6:**
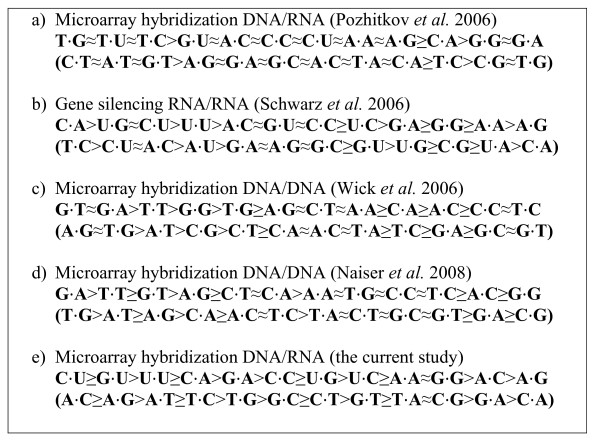
**Stability orders of 12 single-base mismatch types for hybridization in solution (*b*) and on microarrays (*a, c, d, e*)**. For a hybridization oligonucleotides duplex X/Y, X refers to probe and Y refers to target. For example, the microarray hybridization in our study is DNA/RNA, which means the probe is DNA and the target is RNA - cRNA used by an Affymetrix experiment. We refer to Naiser et al. (2008) for details. Specifically, both Wick et al (2006) and our study used log_2_(PM/MM) as the measure of discriminating ability, which has an opposite order compared to stability. Thus, the larger log_2_(PM/MM) value corresponds to the lower stability of duplex. Please note that there are two notations used under each study: the upper one is the notation that is commonly used- for consistency to that in Naiser et al (2008); the lower one is the notation indicating the base change from a perfect matched probe to a mismatched probe - primarily used in the current study. For example, C U in the common notation means the probe provides C and the target provides U for the mismatched base pair, while in the second notation it's A C indicating A is the base on the perfect matched probe and changes to C on the mismatched probe.

We expanded the cross-study comparison of the effect of mismatch type [6] by including the current study (Figure 6). In addition, we quantified the similarities across different studies in terms of the effect of mismatch type (Table 3). We did not include the results of Sugimoto et al (2000) for two reasons: 1. their results were mainly summarized from the analyses of certain trinucleotide stabilities; 2. there was no C-C mismatch type in their study, which would result in a missing value for our comparison. There are several interesting results from this cross-study comparison.

In general, the results from the different studies were only weakly similar (Figure 6, Table 3) in terms of mismatch type. However, there were some areas of agreement. For consistency, we used the same notation for mismatch type as that in Naiser et al (2008) for the illustration of this cross-study comparison (ours was listed as well, see Figure 3 for detail). Both Wick et al's study (2006) and our results placed the G-T mismatch type at a relatively higher position on the stability list- opposite to the discriminating ability list. Both Schwarz et al's study (2006) and our results placed the A-G mismatch type at the last position on the stability list. However, if the comparison is expanded to the group of purine/pyrimidine mismatches instead of individual mismatch types, our study agreed very well with Naiser et al's (2008). When a mismatch occurred to the C-G base pair, a greater loss in signal intensity was observed (Figure 6). However, each of our studies had an outlier, defined as a mismatch type whose real positions are different from expected according to the rules of Watson-Crick base-pairing. Specifically, positions 1 to 6 on the stability list should belong to the mismatch types whose mismatch occurs to the A-T base pair, and positions 7-12 should belong to the mismatch types whose mismatch occurs to the C-G base pair. Naiser et al (2008) reported an A-G mismatch values at a higher position - position 4 - on the stability list than expected, which should have been placed at positions larger than the 6^th ^. Results from the current study indicated a A-A mismatch values at a lower position - position 9 - on the stability list than expected, which should have been placed at positions smaller or equal to the 6^th^. The difference between two studies may be due to the differences in the detailed hybridization conditions and the different microarray platforms used in the two studies --- Naiser et al (2008) designed their own technology for fabricating the chip of microarray while we used the standard Affymetrix GeneChip.

Because both Wick et al (2006) and our study used the same metric - log_2_(PM/MM) - to measure the discriminating ability - opposite to duplex stability. We want to further elaborate the comparison between two studies. Our results were in partial agreement with Wick et al. (Pearson's correlation: 0.47) (Figure 5). We found that, in general, samples with mismatches with probes where the sample is either an A or T had smaller decreases in signal intensity than when the sample contained a G or C which was mismatched with the probe as would be expected from Watson-Crick base-pairing. Wick et al. found that a sample with a C that mismatched the probe had a much greater decrease in signal intensity as compared to a sample with a G that mismatched the probe (the difference in the central region is about three-fold). In contrast, we found no major difference in the amount of the decrease in signal intensity when the mismatch was between a C in the sample or a G in the sample and the probe (they only differ by about 2% in the central region) (Figure 4). It is not clear what caused the discrepancies between our study and Wick et al.'s study. Wick et al.'s results are in good agreement with values of solution studies with nearest neighbor model [17]. However, it has been reported that the stacking free energies on Affymetrix arrays are different from that in solution [4,18]. But there was also study showing a good agreement between the solution nearest-neighbor(NN) affinities and that on Affymetrix arrays [19]. Further experimentation might resolve these differences.

In general, it appears that mismatch type is more sensitive to different experimental methods than mismatch position. Microarrays produced in different ways may have different spatial features that complicate specific types of mismatched base pairing. This may be because mismatch type depends on many different experimental factors, e.g., solid substrate or solution, DNA or RNA (either probe or target) and the length of probe.

### Practical Implementation of Our Analyses to Microarray Design

Our results may be useful for improving future versions of microarray platform design, especially those which involve the single-base mismatches such as the Affymetrix GeneChip. On the one hand, these results showed that mismatch position effect is relatively insensitive to experimental methods, which supports Affymetrix's decision to choose the 13^th ^position when designing PM/MM probe pairs, (although our results showed that the position 12^th ^may cause a slightly bigger loss of signal intensity in mismatches) (Figure 2 &3). On the other hand, the results from different studies showed that mismatch type is more sensitive to experimental methods, implying that Affymetrix may improve the array's design by incorporating these results. For instance, Affymetrix typically uses a number of probe pairs to represent a probeset, e.g., 11 probe pairs for a gene on Human Genome U133 Plus 2.0 Array. Each probe pairs contains a mismatch probe which alters its 13^th ^position compared to a perfect matched probe. These 11 mismatched bases may not necessarily be the same mismatch type. In fact, they very likely belong to different mismatch types. We found that when the experimental method changes, the effects of mismatch type may also change, indicating there may be some unpredictable uncertainty when summarizing the intensities of these 11 probe pairs. Our results may help Affymetrix minimize this type of uncertainty by incorporating the mismatch type effect when designing probe pairs. A simple resolution is to select the same mismatch type for all 11 probe pairs.

Correspondingly, our results may also be useful for improving array data processing algorithms, particularly those considering single base effects, such as GC-RMA [20] and PDNN [4].

## Conclusion

We evaluated the effects of single-base mismatches on Affymetrix microarrays in a large dataset. We found that a mismatch at the center of a probe incurs a greater loss in signal intensity than the mismatches at the ends of the probe. There was a slight asymmetry between the ends of a probe: effects of mismatches at the 3' end of a probe were greater than those at the 5' end. These results were similar to previous research. The results from studies on mismatch type, including ours, were only weak correlated. Our study agreed very well with a recent study when the comparison was widened to the group effect of purine or pyrimidine mismatch type. Our results, together with that of previous studies, provide a comprehensive assessment of the effects of single-base mismatches on microarrays to date and may be useful for improving future versions of microarray platform design and the corresponding data analysis algorithms.

## Funding

This project was supported by a grant from NIH [RR017444] to RBN and by NIH grant number P20 RR016469 from the INBRE Program of the National Center for Research Resources.

## Competing interests

The authors declare that they have no competing interests.

## Authors' contributions

FD designed the method, analyzed the data and drafted the manuscript. MAP helped the data analyses and contributed to the writing of the paper. ERS supervised data collection and contributed to the writing of the paper. LZ contributed to the writing of the paper. RBN organized the project, supervised data collection and contributed to the writing of the paper. All authors have read and approved the final manuscript.
